# Soft Tissue and Bone Tumor Diagnostics: Harnessing the Power of Molecular Techniques

**DOI:** 10.3390/genes14122229

**Published:** 2023-12-17

**Authors:** Fleur Cordier, Liesbeth Ferdinande, Anne Hoorens, Koen Van de Vijver, Jo Van Dorpe, David Creytens

**Affiliations:** 1 Department of Pathology, Ghent University Hospital, Ghent University, 9000 Ghent, Belgium; fleur.cordier@uzgent.be (F.C.); liesbeth.ferdinande@ugent.be (L.F.); anne.hoorens@uzgent.be (A.H.); koen.vandevijver@uzgent.be (K.V.d.V.); jo.vandorpe@uzgent.be (J.V.D.); 2 CRIG, Cancer Research Institute Ghent, Ghent University Hospital, Ghent University, 9000 Ghent, Belgium

**Keywords:** soft tissue tumors, molecular pathology, DNA/RNA-based next-generation sequencing, copy number variation sequencing, fluorescence in situ hybridization, immunohistochemistry, methylation profiling

## Abstract

Since the introduction of new molecular techniques, the diagnostic landscape of soft tissue and bone tumors has expanded greatly over the past few years. The use of new molecular techniques has led to the identification of new genetic alterations and, therefore, to a better understanding of tumorigenesis, tumor detection and classification. Furthermore, methylation profiling has emerged as a classification tool for soft tissue and bone tumors. Molecular pathology also plays an important role in the determination of patient prognosis and in the identification of targets that can be used for targeted therapy. As a result, molecular pathology has gained a more prominent role in the daily practice of the surgical pathologist. This review delves into various molecular techniques applied in the surgical pathology of soft tissue and bone tumors. It highlights their applications through the analysis of five specific cases.

## 1. Introduction

The diagnosis of soft tissue and bone tumors remains a challenge for most pathologists due to the complexity, histological overlap and rarity of these lesions. Histological heterogeneity within one tumor type and the sometimes small size of tissue biopsies are additional factors that complicate the diagnosis. Over the last years, the discovery of molecular alterations and associated diagnostic immunohistochemistry have provided a foothold. Therefore, molecular pathology has been introduced and applied worldwide in the routine workup of many different soft tissue and bone tumor types, leading to integrated histomolecular diagnosis. In fact, specific molecular panels are already widely used for tumor classification and/or therapeutic assessment and planning. However, in daily practice, most biomarkers are used for diagnosis and not as prognostic and/or predictive therapeutic tools.

Broadly, soft tissue and bone tumors can be divided into two major genetic groups: those characterized by specific genetic alterations and simple karyotypes, and those with non-specific genetic alterations and complex unbalanced karyotypes [1]. As the number of potential molecular tests increases and the cost burden becomes uncontrollable, the pathologist must be able to navigate through the molecular testing strategies. In the following sections, we will provide an overview of the various techniques available to pathologists, designed to enhance their capabilities in the histomolecular diagnosis of soft tissue and bone tumors. 

## 2. Molecular Testing for Diagnosis

Immunohistochemistry (IHC) is a widely used technique at the protein level that still takes the lead among soft tissue and bone tumor diagnostics. IHC is easy to apply and has a short turnaround time, at a low cost. In recent years, with the discovery of specific genetic alterations and their immunohistochemical surrogates, tumor diagnosis can be more precise than ever. Already, there exist surrogate markers for multiple gene fusions (e.g., BCOR IHC for *BCOR*-rearranged sarcomas, STAT6 IHC for solitary fibrous tumors, ALK IHC for inflammatory myofibroblastic tumors (IMTs)), gene amplifications/deletions (e.g., MDM2 IHC for atypical lipomatous tumors/well differentiated liposarcomas and dedifferentiated liposarcomas, loss of SMARCB1 (INI1) and SMARCA4 (BRG1) for epithelioid sarcomas and *SMARCB1/SMARCA4*-deficient malignant tumors), epigenetic alterations (e.g., H3K27me3 IHC for malignant peripheral nerve sheath tumors), single-nucleotide variants (e.g., H3.3 G34W IHC for giant cell tumors of bone) and gene expression profiling (DOG1 for gastrointestinal stromal tumors (GISTs), NKX2.2 in Ewing sarcomas) [2,3].

However, we have to keep in mind that there are still pitfalls and limits in the use of IHC, which sometimes may cause great confusion for the pathologist. For example, not all *BCOR*-rearranged sarcomas are positive for BCOR on IHC, and a subset of IMTs are negative for ALK on IHC because they harbor alternative *ROS1* or *RET* gene fusions instead of an *ALK* fusion. Also, when a malignant neoplasm becomes less differentiated, metastasizes or has been treated, it can lose its immunohistochemical characteristics [4]. Because of these limitations, we must sometimes rely on additional molecular techniques in order to make a precise diagnosis (Table 1 provides an overview of the molecular techniques used in soft tissue and bone tumor diagnosis). 

### 2.1. Fluorescence In Situ Hybridization

**Figure 1:**
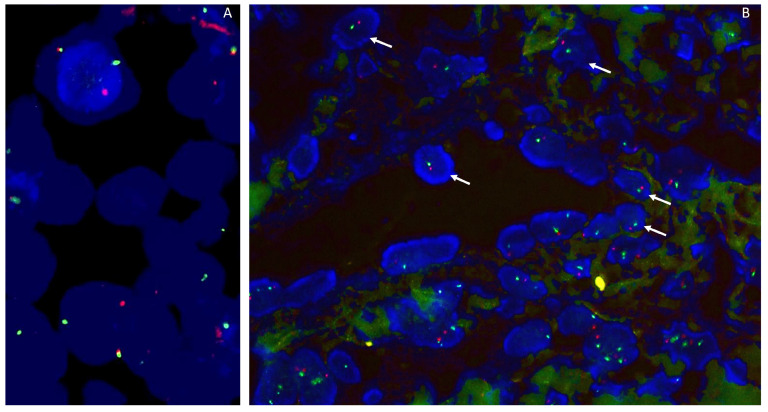
(**A**). FISH SS18 (SYT) using a break-apart probe shows a rearrangement of SS18 in a synovial sarcoma. (**B**). RB1/13q12 Zytovision detection kit, containing two labeled DNA probes. The RB1 probe spans the entire RB1 gene (13q) and is labeled in Spectrum Orange. The 13q12 probe is labeled in Spectrum Green and serves as a control probe. This case shows a monosomy of chromosome 13q (1 red/1 green signal).

Fluorescence in situ hybridization (FISH) is a molecular technique for identifying and locating a specific DNA sequence on a chromosome through the use of specific fluorescence-labeled probes (Figure 1). FISH can be used for the detection of various chromosomal abnormalities in tumors, including gene deletions and amplifications and chromosomal rearrangements. The implementation of FISH on formalin-fixed, paraffin-embedded (FFPE) material is nowadays current practice, with a turnaround time of 1 to 3 days [4]. Moreover, FISH has a resolution at the single-cell level, allowing the evaluation of tumor cells in a background of non-tumoral stromal or inflammatory cells and the assessment of tumor heterogeneity [5]. Still, FISH has its limitations, as it can only target the gene for which the test was designed and is not suited for heavily decalcified material [4,6]. Furthermore, false-negative cases and false-positive cases can occur (see case 3). 

### 2.2. Reverse Transcriptase Polymerase Chain Reaction (RT-PCR), Digital PCR (dPCR) and Multiplex Ligation-Dependent Probe Amplification (MLPA)

Reverse transcriptase polymerase chain reaction (RT-PCR) can be used to detect gene fusions due to gene translocations and can also be performed on FFPE material. It is a very sensitive method that can be used complementarily to FISH for confirmation of results, for equivocal cases or when FISH analysis failed or was not possible. Its similarities with FISH include a short turn-around time (2–4 days) and its inability to detect chromosomal anomalies other than those for which the test was designed [4]. Due to the wide availability of FISH and next-generation sequencing, there has been a reduction in the use of RT-PCR in daily practice. 

Digital PCR (dPCR) is a sensitive and precise molecular detection method widely applied in biomedical fields. Its utility spans across various applications, including trace DNA detection, pathogen identification, rare genetic mutation detection, and determination of copy number variations. Notably, dPCR excels in providing absolute quantification of target nucleic acids, remaining unaffected by contamination that may impact other nucleic acid detection techniques. Therefore, it exhibits higher efficiency and reproducibility compared to RT-PCR [7]. While its high sensitivity is particularly advantageous for detecting genetic mutations in tumor cell subpopulations, a drawback is its high cost [8].

**Figure 2:**
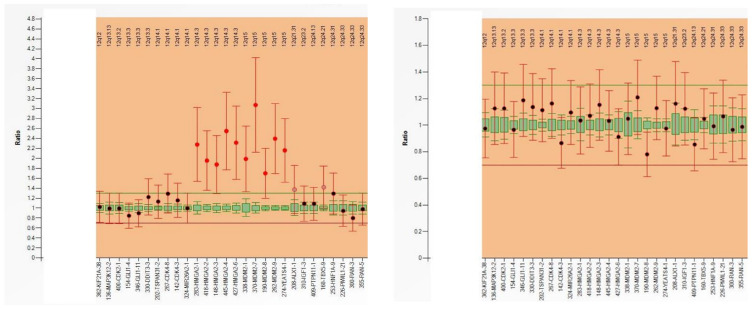
MLPA analysis of a well-differentiated liposarcoma (**left**) reveals MDM2 amplification, whereas a lipoma (**right**) does not exhibit this alteration.

Multiplex ligation-dependent probe amplification (MPLA) is a multiplex PCR method for detecting abnormal copy numbers of up to 50 different genomic DNA sequences (Figure 2). This method allows the determination of the copy number of several different genes in the same PCR and has therefore a low cost. Results can be obtained within 24 h. In contrast to FISH, this technique is less labor-intensive and can be performed on extracted DNA (FISH needs intact tumor cells). One of the main drawbacks of MLPA is that if the percentage of tumor cells is low (due to low tumor cellularity or dilution of tumor DNA with DNA from stromal or inflammatory cells), gain/loss of genomic regions may not be detected (false-negative result) [9,10,11].

### 2.3. Copy Number Variation Sequencing (CNV Sequencing)

**Figure 3:**
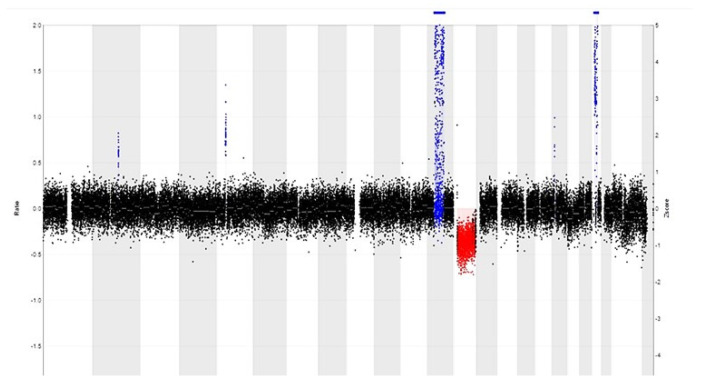
CNV profile of a well-differentiated liposarcoma reveals a relatively simple pattern with 12q amplification (in blue) that includes MDM2 and CDK4 amplification, as well as RB1 gene deletion (in red) at 13q.

Copy number alterations (CNAs) occur in tumors when parts of chromosomes are deleted or amplified. These CNAs can be detected by array comparative genomic hybridization (aCGH). However, the sensitivity of this technique is limited when using FFPE tissue, due to DNA degradation by formalin fixation. This is a disadvantage, since formalin fixation and paraffin embedding is the standard procedure for the examination of surgical biopsies. Also, FFPE material is easier to store than fresh frozen tumor tissue, and its biobanking cost is lower than that of the latter. Furthermore, on FFPE material, areas with a high tumor percentage can be selected for DNA analysis [12]. Recently, copy number variation (CNV) sequencing was introduced to detect CNAs in FFPE material by counting the number of aligned reads within chromosomal windows and by comparing the read counts with the expected number of counts (Figure 3) [12,13]. CNV sequencing is cheaper (when using low-coverage sequencing) than aCGH and has a short turnaround time (7 days). CNV sequencing can be used for the detection of losses/gains at a single locus, e.g., *HER2/Neu* gene amplification in breast tumors, codeletion of *1p* and *19q* in oligodendrogliomas, and of complex chromosomal alterations (large amplifications and/or deletions) [12,14]. In contrast to FISH, this technique is less labor-intensive, less observer-dependent and cheaper when multiple probes are needed. Nevertheless, it should not be neglected that when a tissue has been decalcified, leading to additional DNA damage, interpretations should be made with caution [12].

Somatic CNV evaluation in tumor samples poses bioinformatic challenges due to factors like ploidy, heterogeneity and purity. Further challenges include the difficulty of accurate breakpoint detection, the low resolution of depth of coverage-based methods for small CNVs, and the lack of advanced preprocessing methods. The absence of a CNV gold standard complicates the evaluation. Developing specialized pipelines for NGS data interpretation and CNV identification is laborious, demanding significant time and resources. Ongoing efforts to enhance efficiency, precision, usability and adaptability to tumor complexity are vital, considering their time-intensive nature and associated costs [15]. 

### 2.4. DNA- and RNA-Based Next-Generation Sequencing (DNA/RNA-Based NGS)

Next-generation sequencing (NGS) allows the examination of hundreds to thousands of genes simultaneously in multiple samples and the analysis of different types of genomic features (single-nucleotide variants (SNVs), gene mutations, CNVs, gene fusions, etc.) in a single sequence run, dependent of the design of the test. In comparison with FISH and PCR, this technique has a higher cost and a longer turnaround time. In the diagnosis of soft tissue and bone tumors, NGS can be very helpful, because some gene fusions seem to be tumor-specific (for example, the SS18–SSX gene fusion in synovial sarcoma). However, an increasing number of gene fusions have been shown to be non-histotype-specific and are shared by different tumor types that are otherwise clinically or phenotypically completely unrelated (e.g., the EWSR1–CREB1 gene fusion in angiomatoid fibrous histiocytoma, clear cell sarcoma, hyalinizing clear cell carcinoma of the salivary gland, and primary pulmonary myxoid sarcoma). Therefore, an integrated diagnosis by the surgical pathologist is necessary.

As a result of the application of NGS in daily practice, there is also an increasing number of newly detected fusions, giving rise to a debate about the significance of newly detected fusion partners with, so far, undefined clinical significance [6]. In addition, most pathology laboratories use ‘targeted NGS’, whereby fusions are identified only if one of the gene partners is included in the panel. Nonetheless, NGS is frequently used by pathologists when the diagnosis is not clear at the histological level due to difficulties in differential diagnosis or a small biopsy size, or when there is an unclassifiable histologic morphology or immunohistochemical profile (and FISH is not sufficient). Furthermore, NGS is useful in cases where the fusion partner is relevant for the classification [6].

The highest value for NGS in soft tissue and bone tumor pathology has been demonstrated in cases of monomorphic tumors, round cell sarcomas or spindle cell tumors with a prominent inflammatory infiltrate [6,16]. However, this finding is biased by the selection of probes in the NGS panels, which currently favors the analysis of round cell sarcomas [6].

### 2.5. DNA Methylation Profiling

DNA methylation is an important epigenetic marker and plays an important role in normal development and disease. In cancer, DNA methylation patterns reflect both the cell type of origin and changes acquired during tumor formation. Most studies rely on bisulfite treatment, a method that induces distinct alterations in the DNA sequence depending on the methylation status of individual cytosine residues. The commonly employed assays for diagnostic methylation profiling include the Illumina Infinium 450 K and the EPIC arrays. The Infinium 450 K array assesses the methylation status of 450,000 CpG sites [17], while the EPIC array extends this analysis to 850,000 CpG sites [18]. These high-dimensional methylation profiles can be accurately classified into specific subtypes using machine learning methods, particularly, random forest classification. Additionally, due to the detailed, single-nucleotide resolution information provided by this technique, it is possible to obtain CNV profiles as well [19].

This method is particularly valuable for entities that do not have pathognomonic genetic alterations, such as entity-specific gene fusions. It is also beneficial in cases where identifying a gene fusion through methods like FISH or RNA-based techniques is hindered by technical or specimen-related limitations. This is particularly relevant when dealing with specimens exhibiting prominent crush artifacts or when working with small samples that provide few histologic clues. Methylation profiling can be performed on FFPE material but needs a high tumor cell content, which is difficult to obtain when lesions contain high proportions of non-neoplastic or inflammatory cells or when the DNA is degraded due to the storage conditions. Additionally, it is essential to recognize that the current version of the sarcoma classifier does not encompass the entire spectrum of soft tissue and bone sarcomas but is continuously evolving. Furthermore, interpreting methylation profiling results may not be straightforward [20,21,22,23]. Despite these challenges, methylation profiling remains a valuable tool for sarcoma research and diagnosis, particularly when used in combination with other diagnostic methods. It can provide crucial insights into sarcoma subtypes and aid in improving our understanding of these complex malignancies. Already, there are studies demonstrating the potential of DNA methylation profiling of circulating cell-free DNA (cfDNA) for classifying sarcoma subtypes [20,21].

### 2.6. Nanopore Sequencing

In recent studies, nanopore sequencing was utilized for the ultrarapid sequencing-based diagnosis of sarcomas. This technique has the unique capability to directly detect methylated base pairs without the need for bisulfite modification. This innovative sequencing approach distinguishes between unmethylated cytosine and 5-methylated cytosine by analyzing the distinct ionic current changes produced during sequencing. This technology offers a promising alternative for studying DNA methylation patterns with increased efficiency and accuracy and can be suitable for intraoperative methylation-based tumor classification. Furthermore, the nanopore technology offers the advantage of sequencing long DNA fragments, extending up to approximately 100 kbp. This enables tumor classification based on methylation patterns and chromosomal aberrations. However, low tumor cell content and suboptimal DNA quality can impact the confidence score values associated with these classifications. This method is primarily optimized for fresh tissue samples, and the reference set currently covers only a restricted range of methylation classes [22,24,25].

### 2.7. Liquid Biopsy

Liquid biopsy refers to the sampling and analysis of a patient’s liquids to identify tumor biomarkers by using a variety of molecular methods (PCR, NGS, etc.) on circulating tumor cells, circulating cell-free DNA, circulating exosomes and exosome-associated proteins. The main advantage of this procedure is that it is minimally invasive and therefore can be used widely in a clinical setting. It is also useful in cases where the localization of the tumor poses a risk of complications during biopsy collection. Serum biomarkers have already been used for tumor diagnosis (early detection with assessment of the primary site), prognosis (stage, progression of the tumor, resistance to drugs) and monitoring (recurrence of the tumor), e.g., *BRAF* mutations found in patients with melanoma, exon 11 *KIT* mutations in GISTs, etc. Liquid biopsies also have a potential role in personalized medicine. Their disadvantages are sensitivity issues, a high cost and the (as yet) limited range of biomarkers that can be used, especially when rare tumors are involved (due to the limited number of related studies, which makes it difficult to demonstrate their prognostic value and clinical utility) [26,27]. The characteristics of the tumor also influence the ability to detect biomarkers in liquid biopsies, because the amount of tumor-derived material depends on tumor histology, tumoral cell turnover and tumor burden [27]. Further studies will be necessary to explore whether liquid biopsy is to become part of the diagnostics of soft tissue and bone tumors in the future.

### 2.8. New Evolving Techniques

The use of tumor-educated platelets (TEPs) in liquid biopsies, which harbor specific spliced RNA profiles and may serve as a biomarker, was tested for the diagnosis of sarcoma. The analysis of TEPs showed distinct RNA profiles in liquid biopsies from sarcoma patients compared to those from controls [28].

Another study profiled fragmented cfDNA in liquid biopsies, based on the observation that the fragmentation of DNA from dying tumor cells is not random but seems to reflect the chromatin structure and epigenetic states of the cells from which the DNA fragments originated. Using deep whole-genome sequencing, it was shown that the proportion of short fragments was higher in cfDNA from pediatric patients with Ewing sarcoma compared to cfDNA from controls. This method could be relevant for cancers with little or no genetic alterations or when no histological diagnosis can be obtained [29].

## 3. Molecular Testing for Therapeutic Decision Making

Molecular techniques are used by pathologists for diagnostic purposes, especially in cases with a challenging differential diagnosis of tumors requiring different treatment managements, which makes the test cost-effective. Furthermore, molecular techniques are requested by oncologists for the detection of potentially targetable gene mutations and fusions and the determination of patient eligibility for treatment or inclusion in clinical trials [6]. Recent advances in oncological molecular diagnostics have already led to the identification of clinically useful molecular assays to predict responsiveness or resistance to specific therapies, targeting, for example, *ALK*, *ROS1* or *NTRK* gene fusions [30]. But who or what determines whether a newly found biomarker is useful? When assessing novel targets for the molecular characterization of tumors, priority is typically assigned to those that offer a potential for targetable therapies or have a substantial diagnostic significance. Also, there are crucial criteria that must be fulfilled when applying a tumor biomarker in the standard of care, since, as Hayes stated, “A bad tumor marker is as bad as a bad drug”. Therefore, a tumor biomarker must demonstrate both analytical and clinical validity, along with clinical utility [31]. To ensure that the pathologist maintains focus, it is advisable to adopt an evidence-based approach with careful consideration of the clinical relevance.

## 4. Clinical Applications of Molecular Testing in Soft Tissue and Bone Tumor Pathology

We will illustrate the implementation of different molecular techniques in daily practice by describing five clinical cases. The molecular findings were interpreted together with the histological results to achieve an integrated diagnosis. 

### 4.1. Case 1

**Figure 4:**
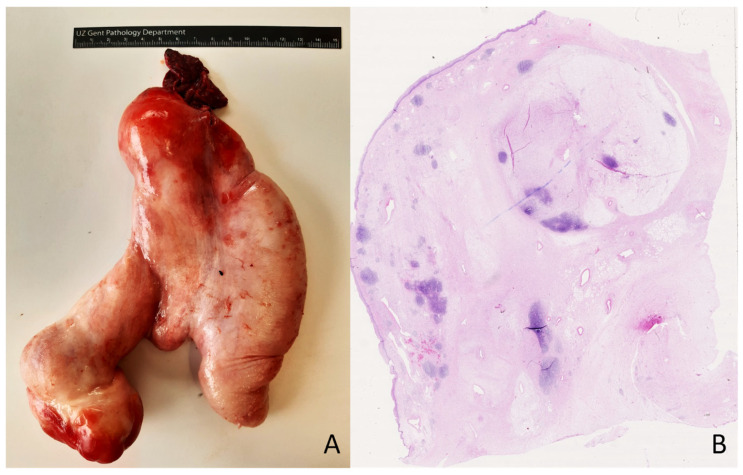
Pedunculated large ‘sausage-shaped’ lesion of the esophagus (**A**). Microscopical overview of the lesion (**B**); hematoxylin and eosin staining, original magnification 10×.

**Figure 5:**
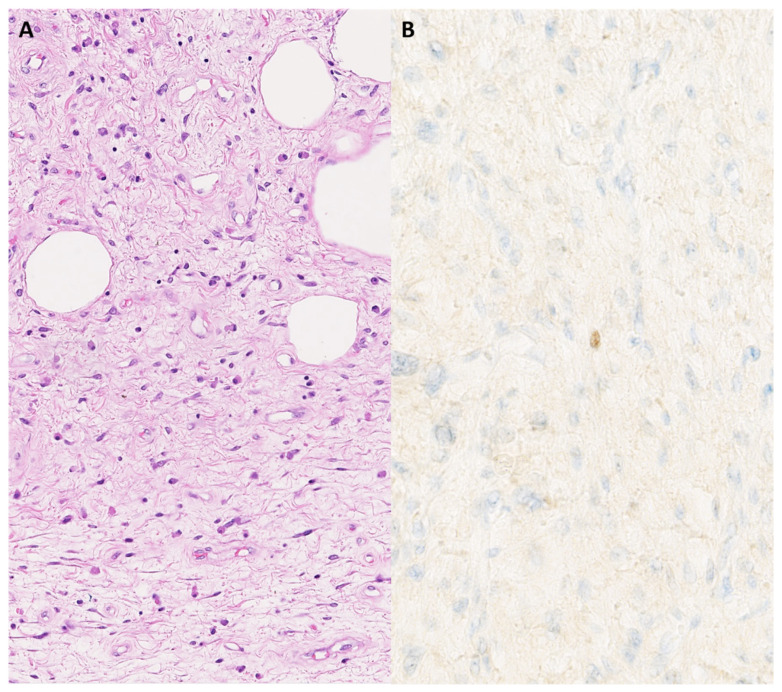
Fibrous lesion with scattered atypical spindle cells and an adipocytic component (**A**); hematoxylin and eosin staining, original magnification 200×. There is focal nuclear MDM2 expression in the atypical stroma cells (**B**); original magnification 400×.

A 54-year-old woman presented with a pedunculated large (18 × 16.5 cm) ‘sausage-shaped’ lesion of the esophagus (Figure 4). The mass had a pink-white color with focal polypoid transformation of the surface. On cross section, it was an encapsulated yellow-white lesion with scattered white-to-brown nodules. Histologically, the resection specimen was lined by squamous epithelium (Figure 4). A subepithelial tumor was observed, composed of a mucinous/myxoid–fibrous stroma with scattered spindle cells with focal cytonuclear atypia. The stromal cells were slightly enlarged, sometimes multinucleated, with irregular nuclei demonstrating granular chromatin (Figure 5). Lymphoid aggregates and lymphoplasmacytic inflammatory infiltrates were scattered in the background. The macroscopically observed nodules showed mature adipose tissue. IHC showed a diffuse expression of p16 and focal nuclear enhancement of MDM2 (Figure 5). *MDM2* FISH was performed, which revealed *MDM2* gene amplification, leading to the diagnosis of a well-differentiated liposarcoma with myxoid and inflammatory morphology. 

Well-differentiated liposarcomas of the esophagus are extremely rare. They present as a large polypoid mass in the lumen of the esophagus and are sometimes mistaken for a ‘giant fibrovascular polyp’. However, careful histologic examination displays adipose tissue intersected by fibrous septa and atypical stromal cells showing MDM2 overexpression by IHC and *MDM2* gene amplification by FISH [32]. The presence of *MDM2* gene amplification, combined with the results from appropriate morphology and immunohistochemistry analyses, is useful diagnostically in the case of a liposarcoma in a rare location and will enable correct diagnosis and management.

### 4.2. Case 2

**Figure 6:**
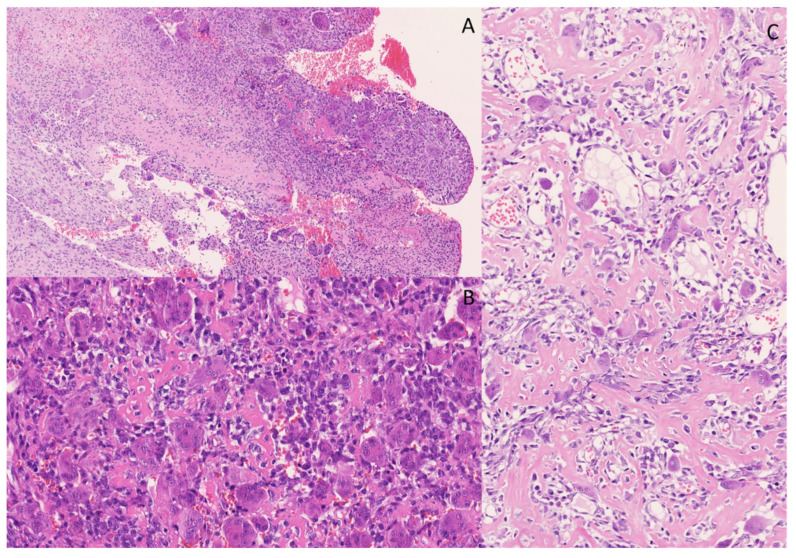
Multicystic lesion with osteoclast-like giant-cells. The cystic spaces are sometimes filled with blood and fibrin (**A**); hematoxylin and eosin staining, original magnification 100×). More solid areas in the lesion were observed, with osteoid, osteoclasts-like giant cells and spindle cells with more pronounced atypia and nuclei varying in size and shape (**B**,**C**); hematoxylin and eosin staining, original magnification 200×).

**Figure 7:**
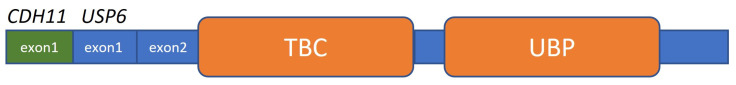
RNA NGS revealed a CDH11::USP6 rearrangement in which exon 1 of CDH11 is fused to exon 1 of USP6. This gene rearrangement positions the complete coding sequence of USP6 downstream of the promoter region of its fusion partner CDH11, leading to transcriptional activation of USP6. USP6 includes a TBC domain (Tre-2/Bub2/Cdc16) and a UBP domain (ubiquitin protease).

A biopsy of a large osteolytic bone lesion in the right temporomandibular joint (caput mandibulae) of a 24-year-old man showed a giant cell-containing tumor with multicystic spaces (Figure 6). Central in the lesion, prominent solid fields with more pronounced cytonuclear atypia and mitotic activity (no atypical mitoses) were present. A lace-like, osteoid and blue bone (basophilic bone) formation was prominent focally. H3.3-G34W staining was negative. CD163 staining was observed in giant cells. SATB2 IHC showed diffuse positivity in the lesion, and MDM2 staining was observed mainly in histiocytes. FISH showed no amplification of the *MDM2* gene. This lesion showed the morphology of an aneurysmal bone cyst (ABC) mixed with solid areas marked by cytonuclear atypia, high mitotic count and bone formation, necessitating the inclusion of a telangiectatic osteosarcoma in the differential diagnosis. Also, its radiological and clinical presentation, as well as the rapid growth of the lesion, were more in favor of an osteosarcoma. Negative H3.3-G34W staining excluded a giant cell tumor of the bone. Targeted RNA sequencing was performed and showed an *USP6::CDH11* gene fusion, which is a highly specific and diagnostic gene fusion for ABC (Figure 7) [33,34].

In this case, differential diagnosis had to be made between ABC and (telangiectatic) osteosarcoma, which are lesions requiring different treatments. Primary ABCs are usually treated by curettage or enucleation and show a recurrence rate of 10%. Osteosarcomas with soft tissue expansion are treated with chemotherapy and surgery and show a much more aggressive behavior [35].

### 4.3. Case 3

**Figure 8:**
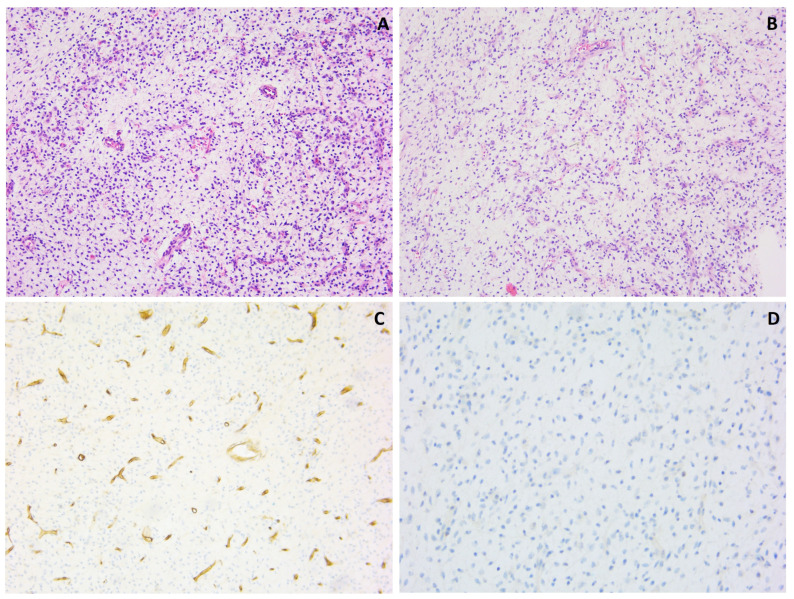
Myxoid lesion with prominent vasculature (**A**,**B**), showing interstitial spindle cells; hematoxylin and eosin staining, original magnification 100×. CD34 staining was negative in the lesional cells and positive in the vessels (**C**); original magnification 100×. There was no expression of MDM2 (**D**); original magnification 200×.

Histological examination of biopsy sections taken from a soft tissue lesion in the thigh of a 31-year-old female revealed a moderately cell-rich lesion with a myxoid/edematous background (Figure 8). There was a prominent vasculature, characterized by numerous intermediate-size fine branching vessels, as well as larger vessels and more thick-walled vessels. No true arcade vessels were observed, but a focal chicken-wire pattern was seen. In the myxoid stroma between the vessels, plump and elongated cells with a slightly vacuolated cytoplasm and indistinct cell borders were found. These cells displayed relatively monotonous nuclei with slight hyperchromatism. Additionally, scattered individual cells with a more prominent vacuolated cytoplasm and large, round-to-oval or bean-shaped nuclei with occasionally prominent eosinophilic nucleoli were observed. Distinct areas with a round cell morphology were not identified. There was no significant mitotic activity, nuclear pleomorphism, or hyperchromasia. On immunohistochemistry, p16 showed a diffuse, strong expression in the lesion. S100 staining highlighted a few individual cells, potentially indicative of dendritic cells. CD34, SMA and desmin staining was negative in the lesion (with positive control staining for CD34 and SMA in the vessels). MUC4 staining was negative, and Rb showed a preserved expression. There was no nuclear expression of MDM2.

Further analysis included FISH *RB1/13q12*, which did not reveal the deletion of the *RB1* gene, and FISH *LSI DDIT3 (12q13)*, which failed to demonstrate a *DDIT3* gene rearrangement.

However, targeted RNA NGS unveiled an *EWSR1::DDIT3* gene fusion, which is pathognomonic and diagnostic for myxoid liposarcoma [36]. The *DDIT3* FISH test was reevaluated, but still did not show convincing evidence of a *DDIT3* gene rearrangement.

Furthermore, immunohistochemistry for DDIT3 was performed and displayed strong nuclear positivity [37]. Taking together the histology and immunohistochemistry results and the presence of a specific *EWSR1::DDIT3* gene fusion, this case should be considered as a myxoid liposarcoma, at least of intermediate grade because of the increased cellularity in this biopsy. A round cell tumor component could not be identified in this material. The negative FISH result may be attributed to a cytogenetically cryptic rearrangement, as the *EWSR1::DDIT3* gene fusion involves more than two double-strand breaks, a configuration that commercial FISH probes may not detect, as described in the literature [36]. This underscores the importance of utilizing multiple levels of analysis in cases with unexpected molecular results.

### 4.4. Case 4

**Figure 9:**
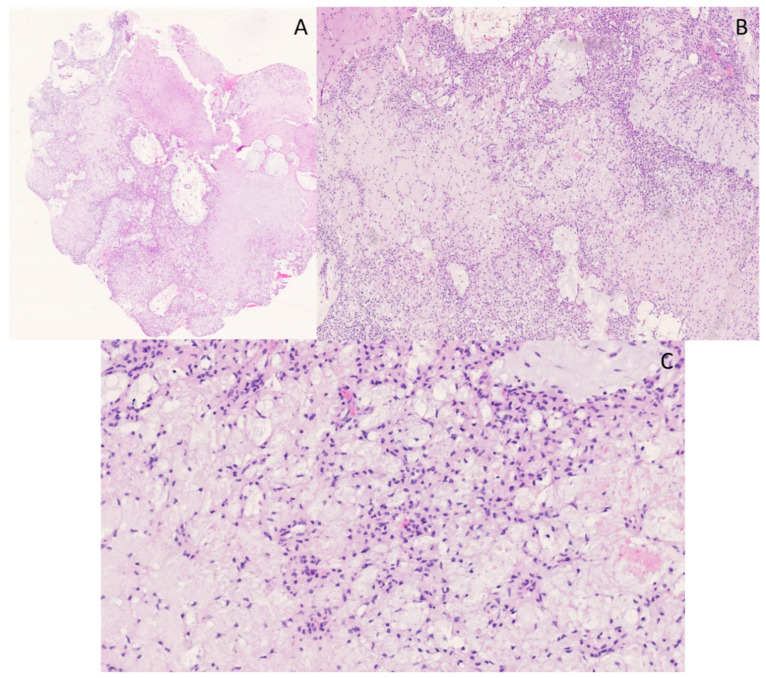
Overview of a lobulated lesion with a chondroid-to-myxoid matrix (**A**); hematoxylin and eosin staining, original magnification 10×. A higher cellularity is seen at the periphery of the lobules (**B**); hematoxylin and eosin staining, original magnification 20×. At a higher magnification, the lesion is composed of a chondroid-to-myxoid matrix with scattered stellate cells with round nuclei and an eosinophilic cytoplasm (**C**); hematoxylin and eosin staining, original magnification, 200×.

The fourth case concerned a lobulated bone lesion at the level of the medial epicondyle of the humerus of a 40-year-old man. The lesion was composed of a chondroid-to-myxoid matrix with scattered stellate cells containing round-to-slightly undulating nuclei and demonstrating an eosinophilic cytoplasm (Figure 9). More cellular areas were intermixed with cell-poor areas. The cellular areas were mainly seen in the periphery of the lobes. IHC showed negativity for MDM2, S100, p63, pancytokeratin AE1/AE3, DOG1 and H3.3-G34W. Based on the morphology, the diagnosis of a chondromyxoid fibroma was made. However, this diagnosis was not entirely consistent with the clinical and radiological presentation (solid lesion in the epicondyle of the humerus with a heterogeneous aspect without level images), which was more in favor of a giant cell tumor of the bone or a chondrosarcoma. The histologic examination did not demonstrate the cytonuclear atypia, mitotic activity and entrapment of surrounding bone typical of a chondrosarcoma. Targeted DNA NGS was performed but did not show *IDH1* or *IDH2* gene mutations, present in 80% of chondrosarcomas [38].

As the chondromyxoid fibroma diagnosis was based on histomorphological observations, without immunohistochemical staining or molecular analyses, the case was referred for a second opinion, due to the clinical and radiological discrepancy. In selected cases, the identification of the upregulation of GRM1 expression in a chondromyxoid fibroma could be a strong diagnostic adjunct in distinguishing this entity from its mimics, but not every pathology laboratory can perform this test [39]. In the end, the diagnosis of a chondromyxoid fibroma was maintained based on the histological appearance of the lesion. This case illustrates that morphology still plays a crucial role in the diagnosis of some soft tissue and bone lesions. 

### 4.5. Case 5

**Figure 10:**
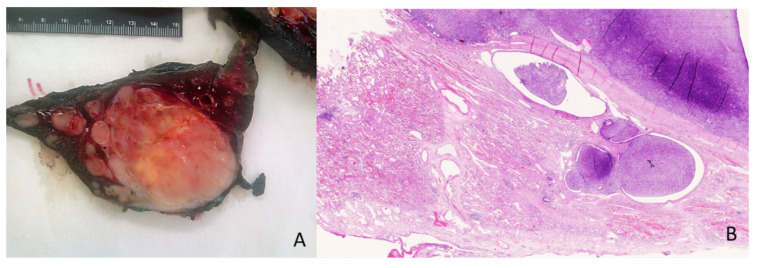
Macroscopic picture of the lesion with a large nodule and surrounding satellite noduli (**A**). Histology showing a main lesion with surrounding satellite lesions, protruding with tongue-like shaped extensions in the airways (**B**); hematoxylin and eosin staining, original magnification 20×.

**Figure 11:**
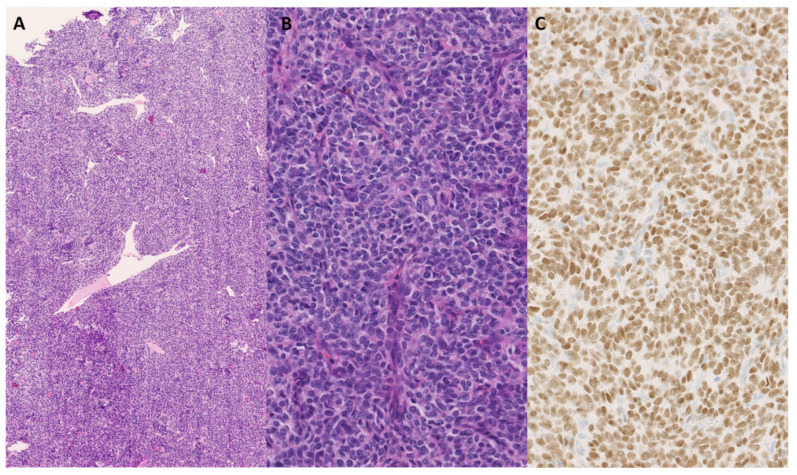
On overview of a cellular lesion with branching, hemangiopericytoma-like vessels wis shown (**A**); hematoxylin and eosin staining, original magnification 100×. The cells were round to oval and epithelioid, with nuclei of varying size and hyperchromatic in appearance, with varying amounts of eosinophilic cytoplasm (**B**); hematoxylin and eosin staining, original magnification 200×. There is strong expression of SATB2 (**C**); original magnification 400×.

A lobectomy specimen from the right lower lobe of the lung of a 64-year-old woman showed a large white tumoral lesion (10 × 6 cm) with surrounding satellite nodules (Figure 10). A microscopic evaluation of the lesion showed sheets and nests of atypical round-to-oval epithelioid cells and spindle-shaped cells with nuclei of varying size, hyperchromatic in appearance, and with varying amounts of slightly eosinophilic and more densely eosinophilic cytoplasm. Some areas in the lesion appeared vaguely pseudopapillary. There was brisk mitotic activity and prominent vascularity, with some dilated and branching vessels. The stroma was collagenous (Figure 11). Morphologically, there was no clearly demonstrable line of differentiation, nor a heterologic tumor component. The lesion showed patchy positivity for pancytokeratin AE1/AE3 and strong expression of SATB2 (Figure 11). A diffuse, cytoplasmic staining for WT1 was observed, with no definite nuclear staining. The lesion was negative for CD34, TTF1, EMA, STAT6, SS18-SSX and TLE1. BCOR staining was cytoplasmic and was therefore considered negative. There was no overexpression of cyclin D1. There was loss of expression of H3K27me2; however, the expression of H3K27me3 was retained. 

The differential diagnosis in this case was broad and included malignant peripheral nerve sheath tumor (MPNST), synovial sarcoma, solitary fibrous tumor (SFT) and *BCOR*-rearranged sarcoma. These entities were, based on IHC, excluded, and the diagnosis of an undifferentiated round cell sarcoma with SATB2 positivity was made. Additionally, targeted RNA NGS was performed, revealing a *JAZF1::SUZ12* gene fusion. This gene fusion is diagnostic for low-grade endometrial stromal sarcoma (ESS) [40]. Cytoplasmic positivity for WT1 and nuclear positivity for SATB2 was described in these tumors [41]. Additional immunohistochemical staining was performed, showing positivity for ER, PR and CD10 and focal positivity for caldesmon. The medical history of the patient mentioned a hysterectomy several years ago, with no information, however, about the presence of a primary tumor. In this case, with an unknown history of a possible primary tumor, the differential diagnosis of an undifferentiated round cell sarcoma was broad, but fortunately molecular testing made a definite diagnosis possible. 

## 5. Conclusions

Diagnosing soft tissue and bone tumors remains complex, and while tissue biopsy serves as the gold standard, achieving a correct integrated diagnosis typically necessitates a combination of morphology, immunohistochemistry, and molecular examinations. This was illustrated in this manuscript through different clinical cases. Over the years, new molecular techniques have been introduced in the diagnosis of soft tissue and bone tumors to bridge existing diagnostic gaps. With advancements in these molecular techniques, there is an increased potential to identify gene fusions of uncertain significance, making it essential to integrate the results from morphological, immunohistochemistry, and clinical and radiological analyses with molecular findings. Additionally, the evolving role of machine learning in tumor classification is contributing to the improvement of diagnostic accuracy. This integrated approach is crucial for achieving accurate diagnoses and optimal clinical treatments, considering the best cost–benefit ratio. Consequently, a multidisciplinary approach remains the standard of care for all soft tissue and bone tumors.

## Figures and Tables

**Table 1:** Overview of the molecular techniques used in the diagnosis of soft tissue and bone tumors.

Technique	Advantages	Disadvantages
**FISH**	- Specific visualization of genetic abnormalities at the chromosomal level - High sensitivity for detecting gene rearrangements, deletions and amplification - Applicable to interphase nuclei in fixed tissues.	- Limited to the targeted regions - Subject to observer variability in interpretation
**RT-PCR, digital-PCR and MLPA**	- High sensitivity and specificity - Digital PCR offers absolute quantification - MLPA allows for multiplex analysis	- RT-PCR is limited to targeted regions - RT-PCR may be affected by amplification biases - Digital PCR may have limited throughput - MLPA is semi-quantitative and may miss novel rearrangements
**CNV sequencing**	- Genome-wide detection of CNVs - High resolution for identifying small variations - Improved sensitivity compared to array-based methods	- Higher cost than targeted approaches - Requires extensive computational analysis - Interpretation complexity due to ploidy, heterogeneity and purity - Limited ability to detect balanced chromosomal rearrangements
**DNA- and RNA-based NGS**	- Comprehensive profiling of genetic alterations - Simultaneous analysis of multiple genes - Detection of novel and known mutations	- High cost and complexity - Bioinformatics challenges in data analysis - Limited ability to detect structural variations
**DNA methylation profiling**	- Epigenetic information for gene expression regulation - Identification of methylation patterns associated with specific soft tissue tumors	- Technical challenges and restricted range of methylation classes - Interpretation complexity due to tissue heterogeneity
**Nanopore sequencing**	- Long-read sequencing for improved structural variant detection - Real-time sequencing without the need for extensive library preparation - Single-molecule sequencing	- Higher error rates compared to short-read technologies - Restricted range of methylation classes - Fresh tissue samples
